# Subarachnoid Hemorrhage Depletes Calcitonin Gene-Related Peptide Levels of Trigeminal Neurons in Rat Dura Mater

**DOI:** 10.3390/cells13080653

**Published:** 2024-04-09

**Authors:** Thannoon Masood, Szandra Lakatos, Gyöngyi Kis, Melissza Ignácz, Ferenc Domoki, Judit Rosta

**Affiliations:** 1 Department of Neurosurgery, Albert Szent-Györgyi Medical School, University of Szeged, Semmelweis utca 6., H-6725 Szeged, Hungary; 2 Department of Physiology, Albert Szent-Györgyi Medical School, University of Szeged, Dóm tér 10., H-6720 Szeged, Hungary; lakatos.szandra@med.u-szeged.hu (S.L.);; 3 Department of Physiology, Anatomy and Neuroscience, Faculty of Science and Informatics, University of Szeged, Közép fasor 52., H-6726 Szeged, Hungary

**Keywords:** meninges, trigeminovascular system, trigeminal ganglion, cerebral vasospasm, headache

## Abstract

Subarachnoid hemorrhage (SAH) remains a major cause of cerebrovascular morbidity, eliciting severe headaches and vasospasms that have been shown to inversely correlate with vasodilator calcitonin gene-related peptide (CGRP) levels. Although dura mater trigeminal afferents are an important source of intracranial CGRP, little is known about the effects of SAH on these neurons in preclinical models. The present study evaluated changes in CGRP levels and expression in trigeminal primary afferents innervating the dura mater 72 h after experimentally induced SAH in adult rats. SAH, eliciting marked damage revealed by neurological examination, significantly reduced the density of CGRP-immunoreactive nerve fibers both in the dura mater and the trigeminal caudal nucleus in the medulla but did not affect the total dural nerve fiber density. SAH attenuated ex vivo dural CGRP release by ~40% and in the trigeminal ganglion, reduced both CGRP mRNA levels and the number of highly CGRP-immunoreactive cell bodies. In summary, we provide novel complementary evidence that SAH negatively affects the integrity of the CGRP-expressing rat trigeminal neurons. Reduced CGRP levels suggest likely impaired meningeal neurovascular functions contributing to SAH complications. Further studies are to be performed to reveal the importance of impaired CGRP synthesis and its consequences in central sensory processing.

## 1. Introduction

In subarachnoid hemorrhage (SAH), bleeding occurs among the meningeal layers, more specifically between the arachnoid membrane and the pia mater. However, the outermost meningeal membrane is external to the arachnoid membrane and the dura mater encephali is also clearly involved in the pathomechanism of SAH. Indeed, a characteristic initial symptom of SAH is a sudden-onset severe headache that often recurs with late complications as well. As the dura mater is the most prominent intracranial pain-sensitive structure [1,2], the presence of a distinctive intense headache implies that this meningeal layer likely contributes to the development of SAH-induced complications.

The trigeminal afferent system is responsible for the sensory innervation of the pain-sensitive intracranial structures, particularly the meninges and the major cerebral arteries [3]. A headache is proved to be associated with the activation of the so-called trigeminovascular system, consisting of the peripheral trigeminal afferents, the innervated cerebral and meningeal vasculature, the trigeminal ganglion (TG), and the trigeminal nucleus caudalis (TNC) in the upper cervical dorsal horn [4]. Activation of trigeminal afferents commonly results in a consequent release of the vasodilator peptide calcitonin gene-related peptide (CGRP) from the trigeminal afferents [5,6]. CGRP is a 37-amino acid peptide expressed in a significant proportion of trigeminal primary sensory neurons; it can be demonstrated to be present in cerebral and meningeal sensory nerves and is also abundantly expressed throughout the central nervous system [7,8]. Increased levels of CGRP are highly associated with headache disorders such as migraines; the importance of CGRP in pain-related research became obvious following the introduction of CGRP antagonists as antimigraine pharmaceuticals [9,10]. The CGRP-release and the consequent dilation of meningeal and cerebral blood vessels are involved in the regulation of cerebral blood flow, showcasing its importance in certain pathological conditions such as ischemia and SAH [11,12].

SAH is associated with high mortality and poor clinical outcomes due to its severe complications, notably delayed cerebral vasospasm (CV). The underlying mechanism of CV is poorly understood; dysregulation of cerebrovascular smooth muscle tone is hypothesized to involve both increased vasoconstrictor and decreased relaxant components [13,14]. A decrease in CGRP levels could lead to the attenuation of a potent vasodilator component that may serve as a compensatory mechanism in certain ischemia-related conditions, tilting the balanced cerebrovascular tone toward vasoconstriction [15]. A reduced level of CGRP-immunoreactivity has been described in cerebral perivascular nerves and human blood vessels after SAH [16]. This decrease in CGRP levels and consequent imbalance of cerebral vasomotor tone have been cited as the main cause of the prolonged vasospasm [17,18]. However, an analogous effect of SAH on CGRP levels in the dura mater encephali has not yet been demonstrated to date.

Recently, meningeal lymphatics were shown to transport erythrocytes derived from SAH, contributing to the restoration of the subarachnoid space and highlighting the major role that dural tissues play in the SAH pathomechanism [19]. Still, it is unclear whether and how SAH affects meningeal neurovascular functions in the framework of the complex trigeminovascular system comprising the meningeal vasculature with their associated axonal projections and the downstream neuronal structures. We hypothesize that SAH triggers a detrimental effect on these regulators of intracranial sensory neurovascular functions. Therefore, the main objective of our study was to investigate the effect of SAH on the CGRP-ergic elements of the trigeminovascular system in the dura mater and in the associated peripheral and central structures in a preclinical rat SAH model.

## 2. Materials and Methods

### 2.1. Surgical Procedure and SAH Induction

We used an intracisternal single-injection SAH model in our experiments to induce experimental SAH [20]. Adult male Wistar rats weighing 350–380 g were used. The animals were housed under controlled conditions (12-h light/dark cycle, 22 ± 2 °C, 50–70% relative humidity) and fed on a standard diet and water ad libitum. Rats underwent surgery under general anesthesia induced by isoflurane (4%). The animals were placed in a stereotactic frame and the atlanto-occipital membrane was exposed by muscle dissection. In total, 200 µL of autologous blood was injected into the cisterna magna with a 26-gauge needle over a period of 5 min according to Solomon et al. [21]. The control animals were either injected with an equal volume of artificial cerebrospinal fluid (aCSF, composed of 119 mM NaCl, 26.2 mM NaHCO_3_, 2.5 mM KCl, 1 mM NaH_2_PO_4_, 1.3 mM MgCl_2_, and 10 mM glucose) or only had their atlanto-occipital membrane perforated without any volume being injected. Rats were placed in a prone head-down tilt position (30°) during the interval of the injection and then were re-positioned horizontally while the needle was kept in position for another 30 min to prevent leakage of the CSF. The anesthesia was stopped 30 min after the injection, the skin was closed with sutures, and the animals were allowed to recover. To minimize the suffering of the animals, 0.05 mg/kg Buprenorphin was administered subcutaneously before and after the surgical procedures. Rats were subjected to the experiments 72 h after the induction of SAH.

Furthermore, untreated control naïve animals were used as absolute controls. The experiments were approved by the Ethical Committee for Animal Care of the University of Szeged (approval ID: XIV./2973/2016) and carried out in accordance with the Directive 2010/63/EU of the European Parliament. All efforts were made to minimize the number of animals used and their suffering.

### 2.2. Retrograde Labeling of Trigeminal Afferents

Fluorogold (FG), a retrograde axonal tracer, was injected intracisternally to localize terminal projections of trigeminal axons exposed to the subarachnoid space. Under general anesthesia, after a skin incision in the middle of the neck and dissection of the neck muscles, FG was administered into the cisterna magna (n = 3). Then, 15 μL of 3% FG dissolved in aCSF was injected in 5 min through a cannula placed in the atlanto-occipital membrane. To avoid leakage of the injected tracer, the needle was left in the cisterna magna for an additional 15 min. The needle was then withdrawn and the incision was closed. Five days after the injection, the animals were perfused transcardially and the upper cervical region of the spinal cord containing the lower brainstem was removed. Frozen tissue sections (20 μm) were cut from the tissue samples by using a cryostat (Leica CM1950-Kryostat, Leica Biosystems Nussloch GmbH, Nussloch, Germany). The sections were observed under a fluorescent microscope equipped with a 400 nm wavelength filter.

### 2.3. Experimental Groups

Experimental group sizes were determined based on sample size calculation using Power and Sample Size 3.043 software (power 80%, α 0.05). Overall, 57 rats were randomly assigned to one of four groups: (1) untreated control animals (CTL, n = 11), (2) perforation of the atlanto-occipital membrane (SHAM; n = 17), (3) intracisternal injection of aCSF (CSF, n = 12), and (4) intracisternal injection of autologous blood (SAH, n = 17). The retrograde labeling experiments were performed by injecting FG intracisternally following perforation of the atlanto-occipital membrane in additional animals (FG, n = 3). Table 1 summarizes the groups of animals that were used. A further two animals were used to verify the SAH model by examining the deposition of blood on the surface of the dissected brain following transcardial perfusion 6 h after SAH induction.

### 2.4. Neurological Examination

A modified Garcia scale was applied to assess the neurological outcome 1, 2, and 3 days following surgery in the SHAM, CSF, and SAH groups. We performed four behavioral tests to evaluate limb symmetry, spontaneous activity, climbing, and forelimb walking and the results of each test were graded from 0 to 3. The neurological condition was scored as follows: 3—no deficit, 2—slightly affected, 1—severely affected, and 0—no function, the rat did not move at all. The overall behavioral test score was calculated as the sum of scored points (0–12).

### 2.5. Immunohistochemistry

For immunohistochemistry, the anesthetized rats were perfused transcardially with 4% paraformaldehyde in phosphate buffer (pH 7.4). The brainstem containing the TNC measuring 6 mm in length, located caudally from the obex, was isolated. Afterward, the skull was cut mid-sagittally, the brain was carefully removed, and the TG was isolated. The dura mater encephali was excised from the skull halves and used as a total preparation.

The isolated TNC and the TG were post-fixed for 2 h in the same fixative and then immersed in 0.1 M PBS containing 30% sucrose at 4 °C for 24 h and cut into 16 µm thick longitudinal sections using a cryostat (Leica CM1950-Kryostat, Leica Biosystems Nussloch GmbH, Nussloch, Germany).

The TG and TNC sections and the dura total preparations were processed for staining with the indirect immunofluorescence technique using a mouse anti-CGRP (1:750, Neuromics, Edina, MN, USA) or rabbit anti-CGRP antibody (1:750, Santa Cruz Biotechnology, Dallas, TX, USA) in combination with a mouse monoclonal antiserum raised against β-tubulin III (1:750, Santa Cruz Biotechnology, Dallas, TX, USA) as primary antibodies. Immunoglobulins labeled with Cy3 and DL488 were used as secondary antibodies (all 1:500, Jackson Immunoresearch Laboratories, Westgrove, PA, USA).

### 2.6. Immunohistochemical Imaging and Image Analysis

Immunohistochemical analyses were performed using confocal fluorescence images taken using a ZEISS LSM 700 microscope with the Zen 2010 software (ZEISS, Oberkochen, Germany). The resolution of confocal images was 0.64 µm/pixel with a 10× objective lens. We captured photos with 20× magnification under the same exposure time and camera gain. Also, we applied the same optical section step (1.2 µm) and thickness for all the sections (Z-stack thickness: 24–26 µm for dura mater preparations). Z-stack images were processed to display maximum projection images, which were used for our data analysis. Captured images were saved in 8-bit grayscale TIF format. For image analysis, ImageJ software 1.54c27 was used [22]. The “Max Entropy” threshold setting was applied.

#### 2.6.1. Image Acquisition and Line Scan Analysis in Dura Mater Preparation

Total dura mater preparations obtained from half of the cranial vault of each animal were used for the immunohistochemical staining. A 0.5 mm^2^ area around the branches of the middle meningeal artery was selected for analysis. Quantification of immunoreactive (IR) peripheral axons was performed by line scan analysis [23]. We defined three lines, each measuring 100 µm in length along the branches of the middle meningeal artery perpendicular to the stream of the axons at a maximum distance of 100 µm from the visible arterial branches. Morphometric analysis of IR nerve fibers has been performed by line scan analysis using the ImageJ. The number of peaks was calculated along the lines and was defined as density (peaks/100 µm). The average of the three density values along the three defined lines was considered to be the mean the mean density of each animal. Tubulin, a widely used marker of peripheral axons, was used to determine the total fiber density by visualizing the entire fiber network. The ratio of CGRP-IR fibers to tubulin-IR fibers was determined as the relative density of CGRP-IR nerves (in %).

#### 2.6.2. Immunohistochemical Analysis in the TG

Two sections containing all three divisions of the TG selected randomly from each animal were analyzed by also using ImageJ. All immunoreactive neurons with apparent nuclei in the selected sections were circled and counted to obtain the total CGRP-IR population. The intensity of CGRP-immunoreactivity was determined by cluster analysis according to a 3-grade scale: +− low intensity, ++− intermediate intensity, and +++− high intensity. The number of neurons with +, ++, and +++ intensity was determined relative to the total CGRP-IR population and their percentage distribution was calculated in each section.

#### 2.6.3. Immunohistochemical Analysis in the TNC

The upper cervical region containing the TNC at a similar level in each animal was used for the immunohistochemical analysis. The appropriate level for analysis was previously determined by retrograde labeling in the FG group of animals. The intensity of CGRP-labeling was determined as density in the dorsal horn laminae I–II using ImageJ. The 8-bit images were thresholded using the Max Entropy method and then three parallel 100 μm lines spaced 200 μm apart were drawn. The number of intercepts along the three lines was calculated (intercepts/100 μm) and the average of the three results was used as the reference value for each animal. Furthermore, the width of the CGRP-immunopositive laminae in the dorsal horn was determined (in μm). The intensively labeled region of interest in the right superficial laminae was demarcated and the width of the lamina in each animal was determined as the average of 10 measurements performed along the entire length of the region of interest.

### 2.7. RNA Extraction and Quantitative Real-Time Polymerase Chain Reaction (qRT-PCR)

The mRNA expression of α-CGRP (also known as calcitonin-related polypeptide alpha, CALCA) in the TG was measured by qRT-PCR analysis. Rats (n = 5/group) were anesthetized with thiopental sodium (150 mg/kg, i.p., Braun, Spain) and decapitated. Both TG were quickly removed from the skull and kept at −80 °C until use. The two TG obtained from each animal were processed together. The total RNA was extracted from the tissues using TriXtract^TM^ reagent (G-Biosciences, St. Louis, MO, USA, cat. no. 786-652) according to the manufacturer’s instructions. Briefly, after homogenization of the tissue samples in TriXtract^TM^ reagent, the RNA content was separated into an aqueous phase with the addition of chloroform. The precipitation with isopropyl-alcohol was followed by a washing with 70 % ethanol and then the RNA pellet was dissolved in RNase-free water. The quantity and quality were verified by using a Genova Nano micro-volume spectrophotometer (Jenway, London, UK) at an optical density of 260 and 260/280 nm, respectively; all samples that were used for further analysis exhibited an absorbance ratio in the range of 1.6–2.0. Equal amounts of the RNA were used to synthesize cDNA in each experiment using an iScript cDNA synthesis kit (Bio-Rad, cat. no. 1708891, Hercules, CA, USA). PCR was carried out in a thermocycler (Bio-Rad CFX96TM Optics Module) preparing triplicates of reactions of 10 µL in volumes using iQ™ SYBR^®^ Green Supermix (Bio-Rad, cat. no. 1708882). A pair of primers (Fw: 5′ AGACAGCCGCATCTTCTTGT-3′ and Rev: 5′-TGATGGCAACAATGTCCACT-3′) previously designed by Bangaru et al. was applied in-house to amplify a 142-bp fragment of the glyceraldehyde 3-phosphate dehydrogenase (GAPDH) mRNA [24]. The expression of GAPDH as a housekeeping gene was determined from the same set of samples to use as an internal normalizer. A further primer pair was designed using the National Centre of Biotechnology Information (NCBI) reference sequence database (https://www.ncbi.nlm.nih.gov/Entrez, accessed on 1 April 2022) to amplify a 90-bp fragment of α-CGRP mRNA (Fw: 5′-GCTGCCCAGATCAAGAGTCA-3′ and Rev: 5′-ACCTGGTGAGCGATGACTTG-3′). As a negative control, RNAse-free water was added instead of cDNA. The threshold cycle values (Ct) were used as reference points for calculating relative gene expression. The comparative Ct method, also known as the Δ∆Ct method [25], was implemented to achieve relative quantification. 2^−Δ∆Ct^ values were used to calculate fold changes in target gene expression using control groups as normalizers.

### 2.8. Measuring CGRP Release from Meningeal Afferents

For measurement of CGRP release from meningeal afferents, the method of hemisected skull preparation was used after Ebersberger et al. [26]. Rats (n = 5-5) were anesthetized with thiopental sodium (150 mg/kg, i.p., Braun, Barcelona, Spain) and decapitated. The skull was split along the midline and the brain was removed. The skulls were placed in synthetic interstitial fluid (SIF, composed of NaCl 135 mM, KCl 5 mM, MgCl_2_ 1 mM, CaCl_2_ 5 mM, glucose 10 mM, and Hepes 10 mM at pH 7.4) for 30 min. After incubation, the hemicranial vaults were filled with 250 μL of SIF and after 5 min, the superfusate was collected to determine the basal CGRP release. After collecting the baseline sample, the SIF was replaced by SIF containing 10 mM KCl three times at 5-min intervals to stimulate the CGRP release. The CGRP content of the samples was determined by the enzyme-linked immunosorbent assay (ELISA) technique. The collected samples were processed using a rat CGRP ELISA kit (Novus Biologicals, Centennial, CO, USA) to measure the CGRP content. The CGRP concentration of the samples was expressed in pg/mL with a detection threshold of ~9 pg/mL. The optical density was measured at 450 nm using a microplate reader (MRX, Dynex, software Revelation 4.22, Chantilly, VR, USA).

### 2.9. Statistics

Statistical analysis was performed by using the Statistica 13 software (StatSoft, Tulsa, OK, USA). After confirming normality and homogeneity of variances, the results were analyzed by the analysis of variance (one-way ANOVA) or ANOVA on Ranks (Kruskal-Wallis). The values are expressed either as median or mean ± standard error of the mean (SEM). Statistical comparisons of the CGRP release and PCR measurements were performed between the SHAM and SAH groups by using the Student’s *t*-test or the Mann–Whitney Rank Sum test, where appropriate. In all tests, a probability level of *p* < 0.05 was considered statistically significant.

## 3. Results

In the following paragraphs, we report compelling complementary evidence that SAH inflicts a major effect on the trigeminovascular system in the dura mater by depleting their CGRP content and expression in an adult rat model of SAH.

### 3.1. Neurological Evaluation of the Single-Injection SAH Model

**Figure 1:**
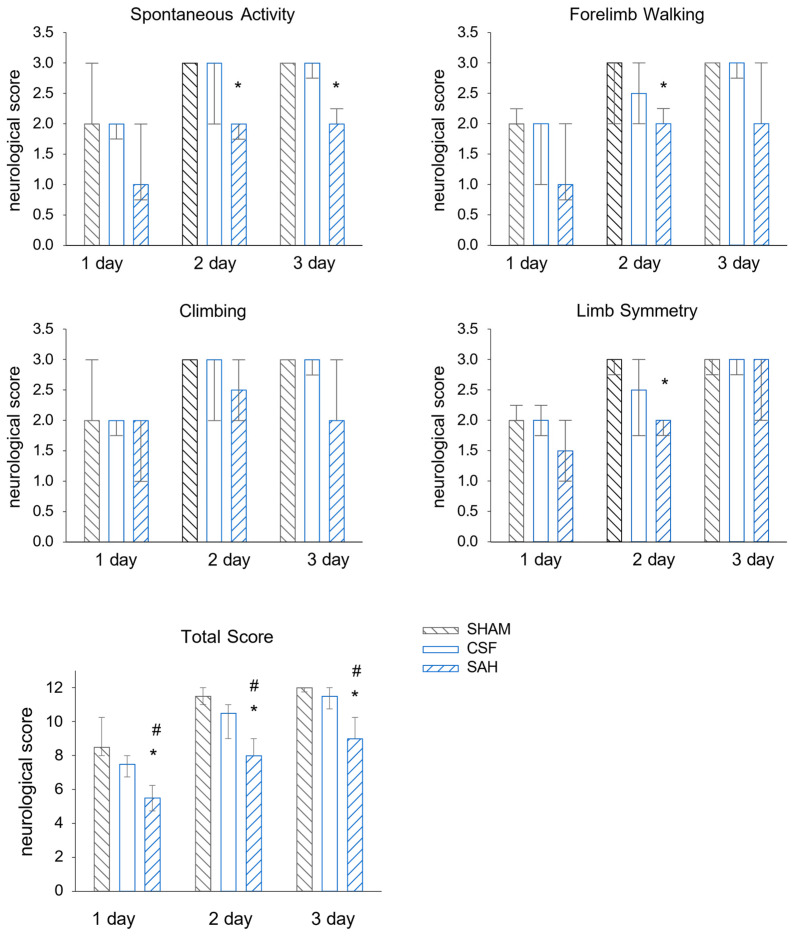
Intracisternal injection of blood worsens neurological outcomes. The results of the four behavioral tests separately (**upper** 4 graphs) and the sum of the scores (graph at the **bottom**). SHAM and CSF animals scored significantly better than SAH animals. All values are expressed as the median with the interquartile range (IQR 25–75%). Data were analyzed by ANOVA on ranks followed by the SNK post hoc test. n = 6 in each group. A probability level of *p* < 0.05 was regarded as statistically significant. *: *p* < 0.05 versus SHAM group, #: *p* < 0.05 versus CSF group.

We tested whether the employed SAH model was efficient enough to inflict neurological deficits. We only observed a small and transient reduction in the neurological scores in the SHAM group at day one, likely reflecting the anesthesia and surgery. Indeed, in the following days, all tested functions were virtually recovered. In the CSF group, the intracisternal injection of aCSF did not cause a significant decrease in the examined neurological functions compared to the SHAM group. The animals in the CSF group were also almost completely recovered in the following days. In contrast, the neurological scores revealed significant SAH-induced brain damage compared to either CSF or SHAM controls at one day post-injection; the median values were 5.5 points vs. 7.3 and 9 points for the SAH vs. the CSF and SHAM groups, respectively (Figure 1). Rats in the SAH group also improved their neurological scores on days two and three (8.3 and 9.5 points, respectively); however, their scores remained significantly lower compared to the animals of both the SHAM and CSF groups.

### 3.2. Macroscopic Inspection of the Brain after SAH

**Figure 2:**
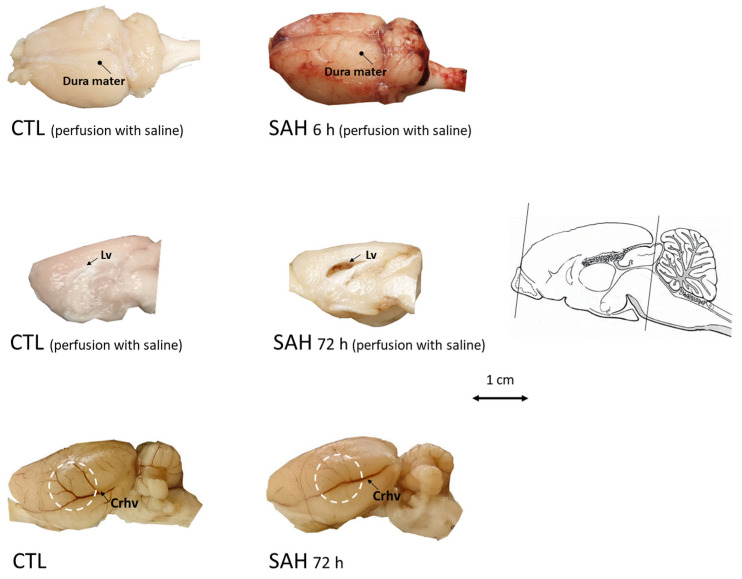
Examination of the brain after SAH induction. **Upper** panel: brains were dissected with the dura mater kept intact after transcardial perfusion with saline in control (CTL, **left**) or 6 h after SAH (SAH, **right**). Marked blood deposition beneath the dura mater on the entire cerebral surface can be readily observed in the SAH animals whereas blood is absent in CTL. **Middle** panel: at 72 h after SAH induction, after transcardial perfusion with saline, blood is visible in the enlarged lateral ventricle (**right**, medial view of the right hemisphere) unlike in CTL (**left**). **Bottom** panel: at 72 h after SAH induction, edema and impaired filling of superficial cerebral blood vessels (**right**, lateral view of unperfused left hemisphere) can be seen compared to the control (no perfusion). Lv: Lateral ventricle, Crhv: Caudal rhinal vein, scale bar arrow shows the rostro-caudal axis.

Examination of the brain showed deposition of the blood at the surface of the brain 6 h after the induction of SAH (Figure 2). In SAH animals, we also observed the signs of edema such as a flattened surface of the brain and enlargement of lateral ventricles. Then, 72 h after SAH, the large superficial cerebral veins became less visible as a sign of venule vasospasm (Figure 2).

### 3.3. SAH Depletes CGRP in Perivascular Nerves in the Rat Dura Mater

**Figure 3:**
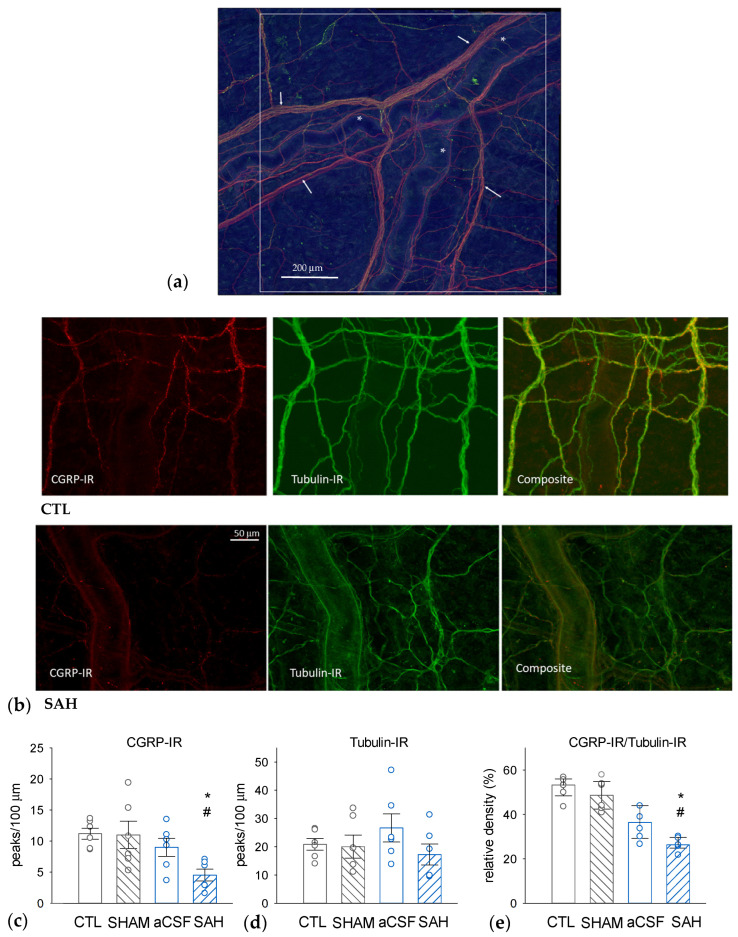
SAH reduces the proportion of CGRP-immunoreactive (IR) nerve fibers in the rat dura mater. (**a**) A representative image—a composite figure of CGRP-IR, red) and the pan-neuronal marker Tubulin-IR (green) immunostaining—showing the dural area around the middle meningeal artery selected for further analysis. The white square represents 1 mm^2^. Asterisks (*) indicate branches of the middle meningeal artery and the arrows indicate Tubulin-IR perivascular nerve fibers. (**b**) Representative photomicrographs were taken from a naïve control (CTL, upper panel) and an SAH animal showing CGRP-IR (**left**), Tubulin-IR (**middle**), and the composite image (**right**). The insets show perivascular CGRP-IR nerve fiber bundles. Densities of CGRP-IR (**c**) and Tubulin-IR (**d**) nerve fibers, as well as CGRP-IR/Tubulin-IR density ratios (**e**). While Tubulin-IR was not significantly different in any experimental groups, CGRP-IR and the CGRP-IR/Tubulin-IR ratios were significantly reduced in the SAH group compared to CTL and sham-injected (SHAM) animals. Data are expressed as mean ± SEM and were analyzed by one-way ANOVA (Tubulin-IR) or expressed as median with range (IQR 25–75%) analyzed by Kruskal-Wallis One Way ANOVA on Ranks (CGRP-IR, and CGRP-IR/Tubulin-IR ratios). Pairwise comparisons were performed with Tukey’s post hoc test in each analysis. *: *p* < 0.05 versus SHAM, #: *p* < 0.05 versus CTL, n = 6 in all groups.

The SAH-induced changes in dural nerve fiber CGRP levels were assessed with quantitative morphometry using immunohistochemical visualization of CGRP-immunoreactive (CGRP-IR) and Tubulin-IR nerve fibers (Figure 3). Tubulin is a pan-neuronal marker that effectively labels all axonal elements. The determination of the densities of CGRP-IR, Tubulin-IR, and their ratios nerve fibers revealed that approximately 50% of the dural nerve fibers are CGRP-positive in naïve CTL animals (Figure 3c–e). Compared to the CTL group, the total density of Tubulin-IR nerve fibers was unaffected in all groups undergoing surgery (Figure 3d). However, the absolute density of CGRP-IR fibers and also the ratio of CGRP-IR to Tubulin-IR fibers were markedly and statistically significantly reduced only in the SAH group. In this group, the ratio of CGRP-positive fibers was reduced to approximately half of the value seen in the CTL and the SHAM groups (Figure 3e). In the CSF group, aCSF injection appeared to slightly but not significantly decrease the relative density of CGRP-immunoreactive fibers but there was also no statistically significant difference between the CSF and SAH groups as well. 

### 3.4. SAH Reduces CGRP Release from the Rat Dura Mater

**Figure 4:**
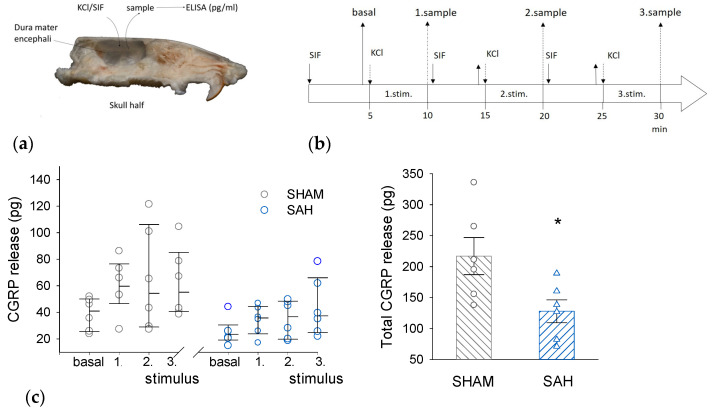
SAH reduces KCl-stimulated CGRP release from the rat dura mater. (**a**) Ex vivo preparation of hemisected skull to measure the CGRP release from the dura mater into the cranial vault filled with synthetic interstitial fluid (SIF). (**b**) Experimental protocol of sample collection. (**c**). Stimulated CGRP release. (**Left**): The amount of released CGRP (in pg/ 250 μL volume of sample) upon the three consecutive stimulations in SHAM and SAH groups. Stimulus-induced CGRP release was analyzed by Kruskal-Wallis One-Way ANOVA on ranks followed by Tukey’s post hoc test. All values were expressed as the median with range (IQR 25–75%), n = 6 in each group. (**Right**): The total amount of released CGRP was expressed as mean ± SEM and the difference between the SHAM and SAH group was analyzed by Student’s *t*-test. n = 6 per group. *: *p* < 0.05.

Basal and KCl-induced ex vivo CGRP release was compared between the SHAM and the SAH group (Figure 4). KCl evoked CGRP release above basal levels in both groups but there was a clear tendency for a higher CGRP release from slightly higher basal levels in the SHAM group versus the SAH group for each KCl stimulation. However, the difference between the two groups became very apparent when the cumulative CGRP release was calculated showing greatly and statistically significantly reduced CGRP release from the dura mater of the SAH group (Figure 4c).

### 3.5. SAH Reduces the Proportion of Intensively CGRP-IR Neuronal Cell Bodies in the TG

**Figure 5:**
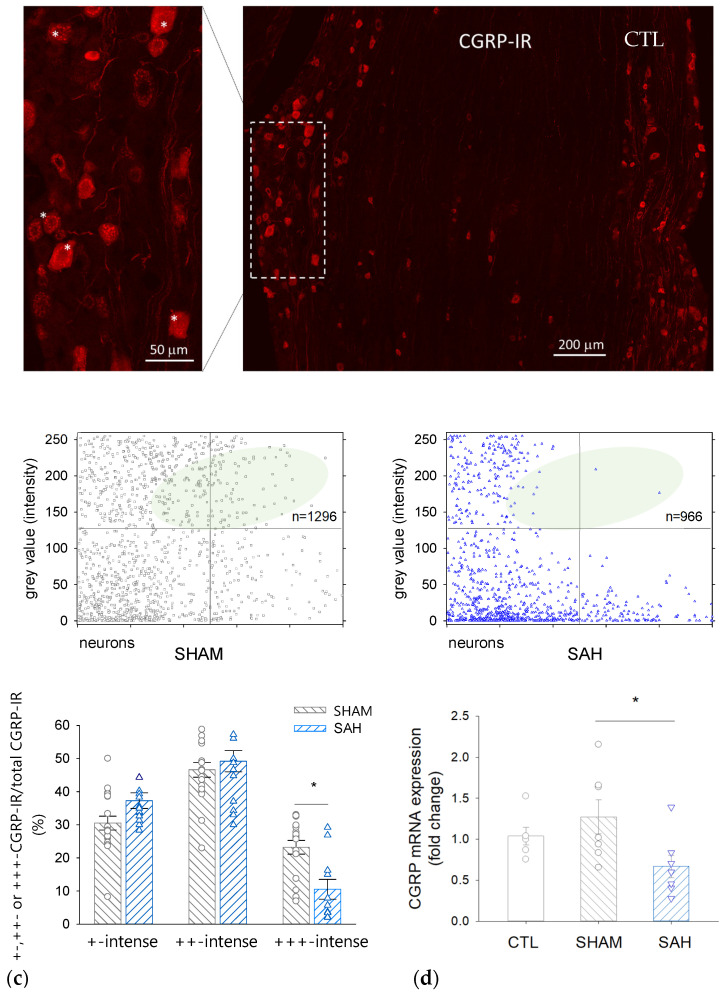
CGRP immunoreactivity and mRNA expression are reduced by SAH in the rat trigeminal ganglion (TG). (**a**) A representative image showing CGRP-immunoreactive cell bodies in the TG of a control rat. Inset: Strong red fluorescence shows +++ CGRP-immunoreactive neurons marked with asterisks. (**b**) Intensity of CGRP-immunoreactivity of individual neurons on a 0–255 grey scale range in SHAM (**left**) and SAH (**right**) animals. The number of high-intensity value cells in the SAH group is reduced. (**c**) Distribution of CGRP-IR neurons according to the staining intensity in SHAM and SAH groups. Data are expressed as mean ± SEM and were analyzed by One-Way ANOVA. n = 7 per each group. *: *p* < 0.05. (**d**) CGRP mRNA expression in the rat TG in the naive control (CTL, SHAM, and SAH groups). Data are expressed as mean ± SEM and were analyzed by One-Way ANOVA followed by Tukey’s post hoc test.

A decrease in CGRP in the perikarya of trigeminal primary sensory neurons was demonstrated by morphometric analysis (Figure 5a–c). SAH reduced the ratio of the most intensively fluorescent CGRP-IR neurons by ~50% compared to the SHAM control group.

Simultaneously, the proportion of less intensively labeled neurons tended to be higher in the SAH compared to SHAM animals (the ratio of ‘+-intense’ cells was 37 ± 6% vs. 31 ± 7% and that of the ‘++-intense’ cells was 49 ± 10% vs. 47 ± 7%, SAH vs. SHAM, respectively). The number of the counted CGRP-IR neurons was between 132–278 in each section and the total number of CGRP-IR cells was 1296 in the SHAM and 966 in the SAH group.

### 3.6. SAH Attenuates CGRP mRNA Expression in the TG

qRT-PCR revealed that CGRP expression in the TG isolated from naïve CTL and SHAM animals was not significantly different; however, it was significantly reduced by approximately 40–50% in SAH animals compared to control groups (Figure 5d).

### 3.7. SAH Depletes CGRP also in the Central Trigeminal Projections in the Trigeminal Nucleus Caudalis (TNC)

**Figure 6:**
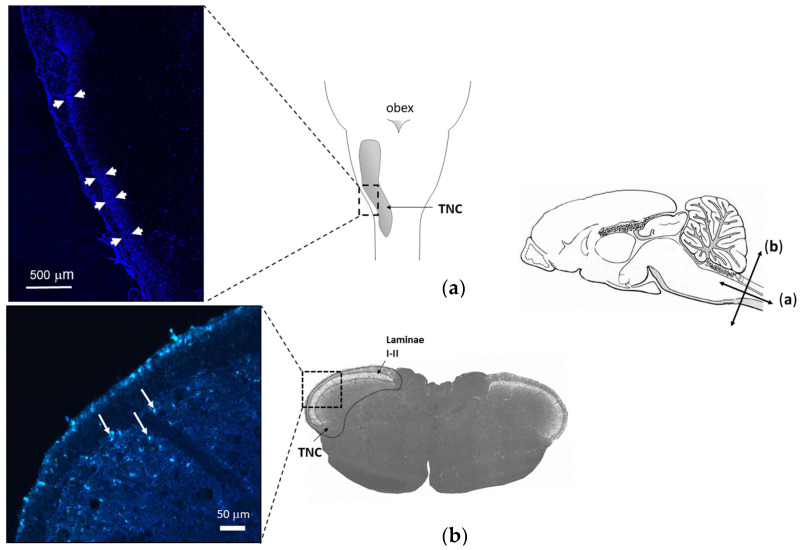
Localization of terminal projections of axons exposed to the subarachnoid space by retrograde labeling. Schematic drawing of the mid-sagittal view of a rat’s brain (**right**). Arrows indicate longitudinal (**a**) and transverse (**b**) scales. (**a**) Longitudinal view of rat brain with TNC. (**Left**): FG labeling (arrows) along the TNC in a longitudinal section (**b**) A representative transverse section including the region of TNC. Magnified image (**left**) shows FG labeling (white arrows) in the superficial laminae 5 days after intracisternal injection of the tracer.

**Figure 7:**
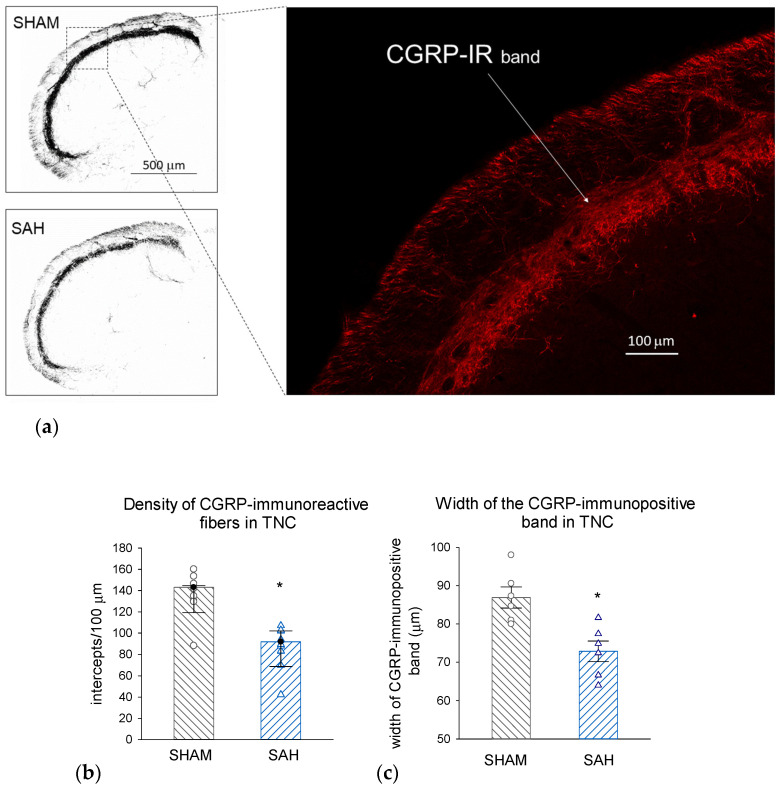
SAH depletes CGRP-IR nerve terminals in the TNC. (**a**) Representative photomicrographs showing CGRP-immunolabeling in the dorsal region of the TNC from a SHAM (**top**) and a SAH (**bottom**) animal. Scale bar: 500 μm. Inset: Magnified section of the dorsal lamina with CGRP-IR nerve fibers (red). Scale bar: 100 μm. (**b**) Density of CGRP-IR fibers was significantly reduced by SAH. Data were analyzed by the Mann–Whitney Rank Sum Test and the values are expressed as medians with range (IQR 25–75%). n = 6. *: *p* < 0.05. (**c**) The thickness of the CGRP-IR dorsal lamina in TNC was significantly narrowed in the SAH compared to the SHAM groups. Data are expressed as mean ± SEM and were analyzed by *t*-test. n = 6, *: *p* < 0.05. TNC: trigeminal nucleus caudalis, FG: fluorogold tracer, CGRP-IR: CGRP-immunoreactive nerve fibers.

FG retrograde labeling identified the central projection of those trigeminal afferents that innervate the meningeal membranes as the caudal region of the TNC (Figure 6). Morphometric analysis of the identified region of interest showed that SAH significantly attenuated the density of CGRP-IR nerve fibers in the TNC compared to SHAM animals; this attenuation also manifested in the significant narrowing of the CGRP-IR band of terminals identifying the appropriate laminae of the TNC (Figure 7).

## 4. Discussion

The major finding of the present study is the novel observation that SAH elicits depletion of CGRP in trigeminal sensory neurons serving the outermost meningeal layer, the dura mater. This finding is in accordance with previous data demonstrating decreased CGRP levels in perivascular nerves on the pial (brain) surface after SAH [27] and lends further support to the hypothesis that decreased CGRP levels contribute to the severe consequences in SAH [28,29] In the present study, we provide morphological and quantitative evidence on the detrimental effects SAH exerts on the trigeminovascular system integrity, more specifically, (1) on the density of CGRP-IR trigeminal afferents in the dura mater and the TNC, (2) on the amount of releasable CGRP from the dura mater, and (3) on the CGRP mRNA expression in the TG.

We have validated our single-injection SAH model by neurological examination and macroscopic investigation of the brain. Evaluation of the neurological outcomes showed that the single intracisternal injection of blood did not result in severe neurological deficits, indicating that the single injection is a mild model of SAH. The mild SAH models were reported to involve moderate hemodynamic disturbances but no severe ischemic brain damage [30]. Although cerebral blood flow measurements were not performed in the present study, the macroscopical examination of the brain showed impaired filling of large cerebral veins, indicating decreased venous reperfusion and venous compression. This finding is emphasized by the fact that venule vasospasm clinically develops during SAH and is considered in the recirculation concept of SAH management [31,32] Deposition of blood was observed on the surface of the brain beneath the dura mater, indicating that blood indeed had entered widespread into the subarachnoid space. Significant enlargement of the ventricles was also observed, which is frequently associated with the presence of SAH [33]. Moreover, the SAH-affected brain showed a flattened surface, as a sign of cerebral edema. Cerebral edema is proven to be highly correlated with elevated intracranial pressure resulting from bleeding into the subarachnoid space [34].

Increased intracranial pressure (ICP) (>20 mmHg) is a common symptom in SAH patients, occurring in acute, subacute, and also delayed phases of hemorrhage [35]. It has been questioned for a long time as to whether the elevated ICP is a consequence of bleeding or whether the presence of blood beneath the arachnoid membrane is more prominent in the pathomechanism of SAH. In the single-blood injection model, the injection of blood or saline generates the same increase in ICP [36] and saline injection also greatly affects cerebral perfusion. In the present study, immunohistochemical data only showed a moderate trend of CGRP decrease in the CSF group compared to the naïve CTL group. Still, these results may suggest that a sudden increase in ICP due to intracisternal injection of a certain volume may affect the integrity of CGRP-containing neural elements irrespective of the composition of the injected fluid. This notion suggested we reconsider the role of the CSF group as a relevant control. In patients with SAH, elevated ICP and oxidative effects derived from blood components occur simultaneously, as the two main pathogenetic factors. The aim of this study was not only to compare the pathological effects of elevated ICP alone but also in the presence of blood in an SAH model. Therefore, the findings in the SAH group were mainly compared to the SHAM as a control group and these comparisons were emphasized in the TG CGRP mRNA expression and dural CGRP-release experiments.

Our study focuses on the late pathophysiological changes associated with delayed CV, which occurs as a severe complication of SAH. The prolonged CV as a predictive factor in ischemia-related clinical syndrome [37] most often occurs on the third day and lasts until 14 days after the onset of SAH. In autologous blood injection rat models, a strong contraction of major arteries was seen between 2 and 7 days with a peak on day 5 after the injection, [38]. As our examinations were performed 72 h post SAH-induction, we can assume that CV was already developing in our model when we investigated the CGRP level in the trigeminal system.

Reduced levels of CGRP have been implicated in the development of CV as preceding studies on SAH revealed that an enhanced release of CGRP resulted in the loss of cerebral perivascular CGRP [28,39]. Furthermore, the amount of CGRP in the CSF of patients was found to be inversely correlated with the severity of vasospasm [40]. CV can persist even months after the SAH in patients [41], indicating long-term pathological changes associated with SAH. Prolonged stimulation of peptidergic afferents may lead to the depletion of CGRP stores, which is hypothesized to account for the loss of CGRP from the nerves surrounding the cerebral vessels [42]. Peptide synthesis in cell bodies is supposed to restore the level of CGRP at the periphery. Three days after SAH, however, we measured a decrease in the expression of CGRP in trigeminal neurons. A reduction in CGRP synthesis in trigeminal neurons is supposed to trigger a negative effect on the restoration of releasable CGRP in peripheral nerve endings, proposing a prolonged attenuation of CGRP action in the trigeminovascular system. Further experiments are needed to elucidate whether such prolonged changes in CGRP levels are indeed involved in the delayed pathological events of SAH.

A recent study observed the narrowing of pial arterioles and emphasized their important contribution to reduced cortical perfusion after SAH [43], suggesting that besides intraparenchymal blood vessels, the meningeal vessels are also involved in the adverse cerebrovascular events associated with SAH. In the present study, we found that SAH resulted in a ~50% reduction in CGRP-containing fibers in the rat dura mater that represent almost half of the sensory fibers in untreated controls. An adequate level of CGRP has been shown recently to be required for the integrity of the meningeal lymphatic system [44] and has a prominent role in the removal of blood from the subarachnoid space after SAH [19]. Therefore, the decreased level of CGRP in the meningeal afferents could impact the pathology of SAH through a deleterious effect on meningeal lymphatic functions.

Stimulation of meningeal afferents results in the activation of second-order neurons in the upper cervical dorsal horn in TNC [45]. CGRP is released both by peripheral and central endings of primary sensory neurons, thus, the stimulation-induced release of CGRP may also affect the second-order neurons of the central nervous system [46]. To examine the changes in the presence of CGRP in the central trigeminal system, we first used retrograde labeling to localize the region of TNC involving terminal endings of meningeal afferents. The intracisternally injected FG was taken up by peripheral endings of those meningeal afferents that are accessible from the subarachnoid space and was transported retrogradely to central endings in the TNC. We found a reduction in the CGRP levels in the corresponding region of TNC, indicating a loss of CGRP also from central endings of meningeal afferents. As CGRP seems to impact the gene expression and synaptic transmission in the TG and the TNC [46], the observed changes in CGRP levels of central trigeminal projections may indicate consequences affecting the central processing of sensory signaling originating in intracranial structures well beyond the direct vasomotor responses.

To the best of our knowledge, this is the first study to demonstrate a decrease in CGRP levels in dural afferents in an SAH model, proposing the role of meningeal afferents in the pathomechanism of SAH. Furthermore, we showed modified expression of CGRP and its decreased level both centrally and in the periphery, supposing delayed consequences in sensory neurovascular functions. This finding may provide further insight into the pathomechanism of CV and the underlying cause of the decrease in cerebral perivascular CGRP.

## 5. Conclusions

Our results confirm that the outermost meningeal layer, the dura mater encephali, is affected during an SAH event, as hemorrhage causes a decrease in the CGRP content of the meningeal perivascular nerves. The release of CGRP from meningeal afferents and the accompanying dilation of meningeal vessels is a well-known phenomenon but its significance has not yet been elucidated. The demonstrated decrease in the amount of CGRP in both the central and peripheral elements of the trigeminal system supposes a prolonged deficit of CGRP-mediated meningeal neurovascular functions after bleeding. We propose that insufficient meningeal neurovascular functions due to reduced levels of CGRP should be considered in future SAH studies, as this may be a significant factor in the clinical outcome after SAH.

## Figures and Tables

**Table 1:** Groups of experimental animals.

Experimental Group	CTL	SHAM	aCSF	SAH	FG
surgical procedure	-	+	+	+	+
intracisternal injection	-	-	200 μL aCSF	200 μL blood	15 μL FG in aCSF
survival time	-	72 h	72 h	72 h	5 days

CTL: untreated control, aCSF: artificial cerebrospinal fluid, CSF: aCSF-injected group, SAH: subarachnoid hemorrhage group, FG: fluorogold tracer.

## Data Availability

The original contributions presented in the study are included in the article; further inquiries can be directed to the corresponding authors.

## Abbreviations

CGRP	calcitonin gene-related peptide
CSF	cerebrospinal fluid
CV	cerebral vasospasm
ELISA	Enzyme-linked Immunosorbent Assay
ICP	intracranial pressure
IR	immuno-reactive
qRT-PCR	Quantitative Real-Time Polymerase Chain Reaction
SEM	standard error of the mean
SIF	synthetic interstitial fluid
SAH	subarachnoid hemorrhage
TNC	trigeminal nucleus caudalis

## References

[1] Feindel W., Penfield W., McNaughton F. (1960). The tentorial nerves and Iocalization of intracranial pain in man. Neurology.

[2] Wirth F.P., Van Buren J.M. (1971). Referral of pain from dural stimulation in man. J. Neurosurg..

[3] Mayberg M., Langer R.S., Zervas N.T., Moskowitz M.A. (1981). Perivascular meningeal projections from cat trigeminal ganglia: Possible pathway for vascular headaches in man. Science.

[4] May A., Goadsby P.J. (1999). The trigeminovascular system in humans: Pathophysiologic implications for primary headache syndromes of the neural influences on the cerebral circulation. J. Cereb. Blood Flow Metab..

[5] Goadsby P.J., Edvinsson L., Ekman R. (1988). Release of vasoactive peptides in the extracerebral circulation of humans and the cat during activation of the trigeminovascular system. Ann. Neurol..

[6] Zagami A.S., Goadsby P.J., Edvinsson L. (1990). Stimulation of the superior sagittal sinus in the cat causes release of vasoactive peptides. Neuropeptides.

[7] O’Connor T.P., van der Kooy D. (1988). Enrichment of a vasoactive neuropeptide (calcitonin gene related peptide) in the trigeminal sensory projection to the intracranial arteries. J. Neurosci..

[8] Moskowitz M.A., Buzzi M.G., Sakas D.E., Linnik M.D. (1989). Pain mechanisms underlying vascular headaches. Progress Report 1989. Rev. Neurol..

[9] Edvinsson L., Goadsby P.J. (1994). Neuropeptides in migraine and cluster headache. Cephalalgia Int. J. Headache.

[10] Naghdi S., Underwood M., Madan J., Brown A., Duncan C., Matharu M., Aksentyte A., Davies N., Rees S., Cooklin A. (2023). Clinical effectiveness of pharmacological interventions for managing chronic migraine in adults: A systematic review and network meta-analysis. J. Headache Pain.

[11] Burnstock G. (1990). Local mechanisms of blood flow control by perivascular nerves and endothelium. J. Hypertens. Suppl..

[12] Wahl M., Schilling L., Parsons A.A., Kaumann A. (1994). Involvement of calcitonin gene-related peptide (CGRP) and nitric oxide (NO) in the pial artery dilatation elicited by cortical spreading depression. Brain Res..

[13] Lenz I.J., Plesnila N., Terpolilli N.A. (2021). Role of endothelial nitric oxide synthase for early brain injury after subarachnoid hemorrhage in mice. J. Cereb. Blood Flow Metab..

[14] Hamann G., Isenberg E., Strittmatter M., Schimrigk K. (1993). Absence of elevation of big endothelin in subarachnoid hemorrhage. Stroke.

[15] Nozaki K., Uemura Y., Okamoto S., Kikuchi H., Mizuno N. (1989). Relaxant effect of calcitonin gene-related peptide on cerebral arterial spasm induced by experimental subarachnoid hemorrhage in dogs. J. Neurosurg..

[16] Edvinsson L., Ekman R., Jansen I., McCulloch J., Mortensen A., Uddman R. (1991). Reduced levels of calcitonin gene-related peptide-like immunoreactivity in human brain vessels after subarachnoid haemorrhage. Neurosci. Lett..

[17] Inoue T., Shimizu H., Kaminuma T., Tajima M., Watabe K., Yoshimoto T. (1996). Prevention of cerebral vasospasm by calcitonin gene-related peptide slow-release tablet after subarachnoid hemorrhage in monkeys. Neurosurgery.

[18] Sun B., Shen F., Wu Q., Chi S., Yang M., Yuan H., Xie F., Zhang Y., Chen J., Zhang F. (2010). Intranasal delivery of calcitonin gene-related peptide reduces cerebral vasospasm in rats. Front. Biosci. Elite Ed..

[19] Chen J., Wang L., Xu H., Xing L., Zhuang Z., Zheng Y., Li X., Wang C., Chen S., Guo Z. (2020). Meningeal lymphatics clear erythrocytes that arise from subarachnoid hemorrhage. Nat. Commun..

[20] Wajima D., Sato F., Kawamura K., Sugiura K., Nakagawa I., Motoyama Y., Park Y.-S., Nakase H. (2017). Venous or arterial blood components trigger more brain swelling, tissue death after acute subdural hematoma compared to elderly atrophic brain with subdural effusion (SDE) model rats. Brain Res..

[21] Solomon R.A., Antunes J.L., Chen R.Y., Bland L., Chien S. (1985). Decrease in cerebral blood flow in rats after experimental subarachnoid hemorrhage: A new animal model. Stroke.

[22] Schneider C.A., Rasband W.S., Eliceiri K.W. (2012). NIH Image to ImageJ: 25 years of image analysis. Nat. Methods.

[23] Gundersen H.J., Bagger P., Bendtsen T.F., Evans S.M., Korbo L., Marcussen N., Møller A., Nielsen K., Nyengaard J.R., Pakkenberg B. (1988). The new stereological tools: Disector, fractionator, nucleator and point sampled intercepts and their use in pathological research and diagnosis. APMIS Acta Pathol. Microbiol. Immunol. Scand..

[24] Bangaru M.L.Y., Park F., Hudmon A., McCallum J.B., Hogan Q.H. (2012). Quantification of gene expression after painful nerve injury: Validation of optimal reference genes. J. Mol. Neurosci. MN.

[25] Livak K.J., Schmittgen T.D. (2001). Analysis of relative gene expression data using real-time quantitative PCR and the 2(-Delta Delta C(T)) Method. Methods San Diego Calif.

[26] Ebersberger A., Averbeck B., Messlinger K., Reeh P.W. (1999). Release of substance P, calcitonin gene-related peptide and prostaglandin E2 from rat dura mater encephali following electrical and chemical stimulation in vitro. Neuroscience.

[27] Nozaki K., Kikuchi H., Mizuno N. (1989). Changes of calcitonin gene-related peptide-like immunoreactivity in cerebrovascular nerve fibers in the dog after experimentally produced subarachnoid hemorrhage. Neurosci. Lett..

[28] Juul R., Aakhus S., Björnstad K., Gisvold S.E., Brubakk A.O., Edvinsson L. (1994). Calcitonin gene-related peptide (human alpha-CGRP) counteracts vasoconstriction in human subarachnoid haemorrhage. Neurosci. Lett..

[29] Imaizumi S., Shimizu H., Ahmad I., Kaminuma T., Tajima M., Yoshimoto T. (1996). Effect of calcitonin gene-related peptide on delayed cerebral vasospasm after experimental subarachnoid hemorrhage in rabbits. Surg. Neurol..

[30] Sun Y., Shen Q., Watts L.T., Muir E.R., Huang S., Yang G.-Y., Suarez J.I., Duong T.Q. (2016). Multimodal MRI characterization of experimental subarachnoid hemorrhage. Neuroscience.

[31] Uhl E., Lehmberg J., Steiger H.-J., Messmer K. (2003). Intraoperative detection of early microvasospasm in patients with subarachnoid hemorrhage by using orthogonal polarization spectral imaging. Neurosurgery.

[32] Zhang J.H. (2014). Vascular Neural Network in Subarachnoid Hemorrhage. Transl. Stroke Res..

[33] Poca M.A., Sahuquillo J., Mataró M., Benejam B., Arikan F., Báguena M. (2005). Ventricular enlargement after moderate or severe head injury: A frequent and neglected problem. J. Neurotrauma.

[34] Shigeno T., Fritschka E., Brock M., Schramm J., Shigeno S., Cervoś-Navarro J. (1982). Cerebral edema following experimental subarachnoid hemorrhage. Stroke.

[35] Alotaibi N.M., Wang J.Z., Pasarikovski C.R., Guha D., Al-Mufti F., Mamdani M., Saposnik G., Schweizer T.A., Macdonald R.L. (2017). Management of raised intracranial pressure in aneurysmal subarachnoid hemorrhage: Time for a consensus?. Neurosurg. Focus.

[36] Kamp M.A., Dibué M., Sommer C., Steiger H.-J., Schneider T., Hänggi D. (2014). Evaluation of a Murine Single-Blood-Injection SAH Model. PLoS ONE.

[37] Kaye A.H., Tagari P.C., Teddy P.J., Adams C.B., Blaso W.P., Boullin D.J. (1984). CSF smooth-muscle constrictor activity associated with cerebral vasospasm and mortality in SAH patients. J. Neurosurg..

[38] Winslow N., Ehsan M., Klopfenstein J. (2022). Delayed ischemic neurologic deficit with vasospasm in aneurysmal subarachnoid hemorrhage after negative post-bleed day 7 angiography. Clin. Neurol. Neurosurg..

[39] Juul R., Edvinsson L., Gisvold S.E., Ekman R., Brubakk A.O., Fredriksen T.A. (1990). Calcitonin gene-related peptide-LI in subarachnoid haemorrhage in man. Signs of activation of the trigemino-cerebrovascular system?. Br. J. Neurosurg..

[40] Schebesch K.-M., Herbst A., Bele S., Schödel P., Brawanski A., Stoerr E.-M., Lohmeier A., Kagerbauer S.M., Martin J., Proescholdt M. (2013). Calcitonin-gene related peptide and cerebral vasospasm. J. Clin. Neurosci..

[41] Kondziolka D., Bernstein M., Spiegel S.M., ter Brugge K. (1988). Symptomatic arterial luminal narrowing presenting months after subarachnoid hemorrhage and aneurysm clipping. J. Neurosurg..

[42] Johansson S., Abdolalizade B., Sheykhzade M., Edvinsson L., Sams A. (2019). Vascular pathology of large cerebral arteries in experimental subarachnoid hemorrhage: Vasoconstriction, functional CGRP depletion and maintained CGRP sensitivity. Eur. J. Pharmacol..

[43] Schwarting J., Nehrkorn K., Liu H., Plesnila N., Terpolilli N.A. (2021). Role of Pial Microvasospasms and Leukocyte Plugging for Parenchymal Perfusion after Subarachnoid Hemorrhage Assessed by In Vivo Multi-Photon Microscopy. Int. J. Mol. Sci..

[44] Mikhailov N., Virenque A., Koroleva K., Eme-Scolan E., Teleman M., Abdollahzadeh A., Giniatullina R., Gafurov O., Krivoshein G., Malm T. (2022). The role of the meningeal lymphatic system in local meningeal inflammation and trigeminal nociception. Sci. Rep..

[45] Strassman A.M., Mineta Y., Vos B.P. (1994). Distribution of fos-like immunoreactivity in the medullary and upper cervical dorsal horn produced by stimulation of dural blood vessels in the rat. J. Neurosci..

[46] Messlinger K. (2018). The big CGRP flood—Sources, sinks and signalling sites in the trigeminovascular system. J. Headache Pain.

